# Epstein–Barr Virus in Gliomas: Cause, Association, or Artifact?

**DOI:** 10.3389/fonc.2018.00123

**Published:** 2018-04-20

**Authors:** Saghir Akhtar, Semir Vranic, Farhan Sachal Cyprian, Ala-Eddin Al Moustafa

**Affiliations:** ^1^College of Medicine, Qatar University, Doha, Qatar; ^2^Biomedical Research Centre, Qatar University, Doha, Qatar; ^3^Oncology Department, McGill University, Montreal, QC, Canada

**Keywords:** brain cancer, glioma, glioblastoma multiforme, Epstein–Barr virus, oncogenesis

## Abstract

Gliomas are the most common malignant brain tumors and account for around 60% of all primary central nervous system cancers. Glioblastoma multiforme (GBM) is a grade IV glioma associated with a poor outcome despite recent advances in chemotherapy. The etiology of gliomas is unknown, but neurotropic viruses including the Epstein–Barr virus (EBV) that is transmitted *via* salivary and genital fluids have been implicated recently. EBV is a member of the gamma herpes simplex family of DNA viruses that is known to cause infectious mononucleosis (glandular fever) and is strongly linked with the oncogenesis of several cancers, including B-cell lymphomas, nasopharyngeal, and gastric carcinomas. The fact that EBV is thought to be the causative agent for primary central nervous system (CNS) lymphomas in immune-deficient patients has led to its investigations in other brain tumors including gliomas. Here, we provide a review of the clinical literature pertaining to EBV in gliomas and discuss the possibilities of this virus being simply associative, causative, or even an experimental artifact. We searched the PubMed/MEDLINE databases using the following key words such as: glioma(s), glioblastoma multiforme, brain tumors/cancers, EBV, and neurotropic viruses. Our literature analysis indicates conflicting results on the presence and role of EBV in gliomas. Further comprehensive studies are needed to fully implicate EBV in gliomagenesis and oncomodulation. Understanding the role of EBV and other oncoviruses in the etiology of gliomas, would likely open up new avenues for the treatment and management of these, often fatal, CNS tumors.

## Introduction

### Gliomas and Glioblastoma Multiforme (GM)

Gliomas (glial tumors) are the most common malignant brain tumors and account for about 60% of all primary central nervous system (CNS) cancers (1). Around 23,880 new cases of primary CNS tumors are expected to be diagnosed in the United States in 2018 (2). Although rare—accounting for approximately 1.4% of all cancers (3)—they generally have a poor prognosis that leads to a disproportionately high morbidity (patients often exhibit compromised basic and critical functions such as movement and speech) and high mortality (CNS tumors are 10th leading cause of death in the USA) (1). The 5-year survival rate for primary malignant brain and CNS tumors is the sixth lowest among all types of cancers after pancreatic, liver and intrahepatic bile duct, lung, esophageal, and stomach, making gliomas some of the most devastating types of cancers (2). Gliomas originate from astrocytes, oligodendrocytes, and ependymal cells and are consequently classified as astrocytomas, oligodendrogliomas, or ependymomas, respectively (4). According to the World Health Organization (WHO) criteria, gliomas are histologically graded into four grades (grade I–IV). Tumor grading correlates well with tumor morphology, biology, and prognosis.

Glioblastoma multiforme, a fatal grade IV glioma, is the most common glial tumor (accounting for 50–60% of all gliomas) and has the worst prognosis with a median survival of 12–15 months and a 5-year survival rate of less than 5% in adults and 16% in children (5–8). The current standard-of-care includes surgical reduction of the tumor mass following craniotomy and then radiation and chemotherapy with temozolomide (9). GBM is morphologically characterized by increased cellularity, marked nuclear atypia, abundant mitotic activity of neoplastic cells followed by the neoangiogenesis, and/or tumor necrosis.

**Figure 1:**
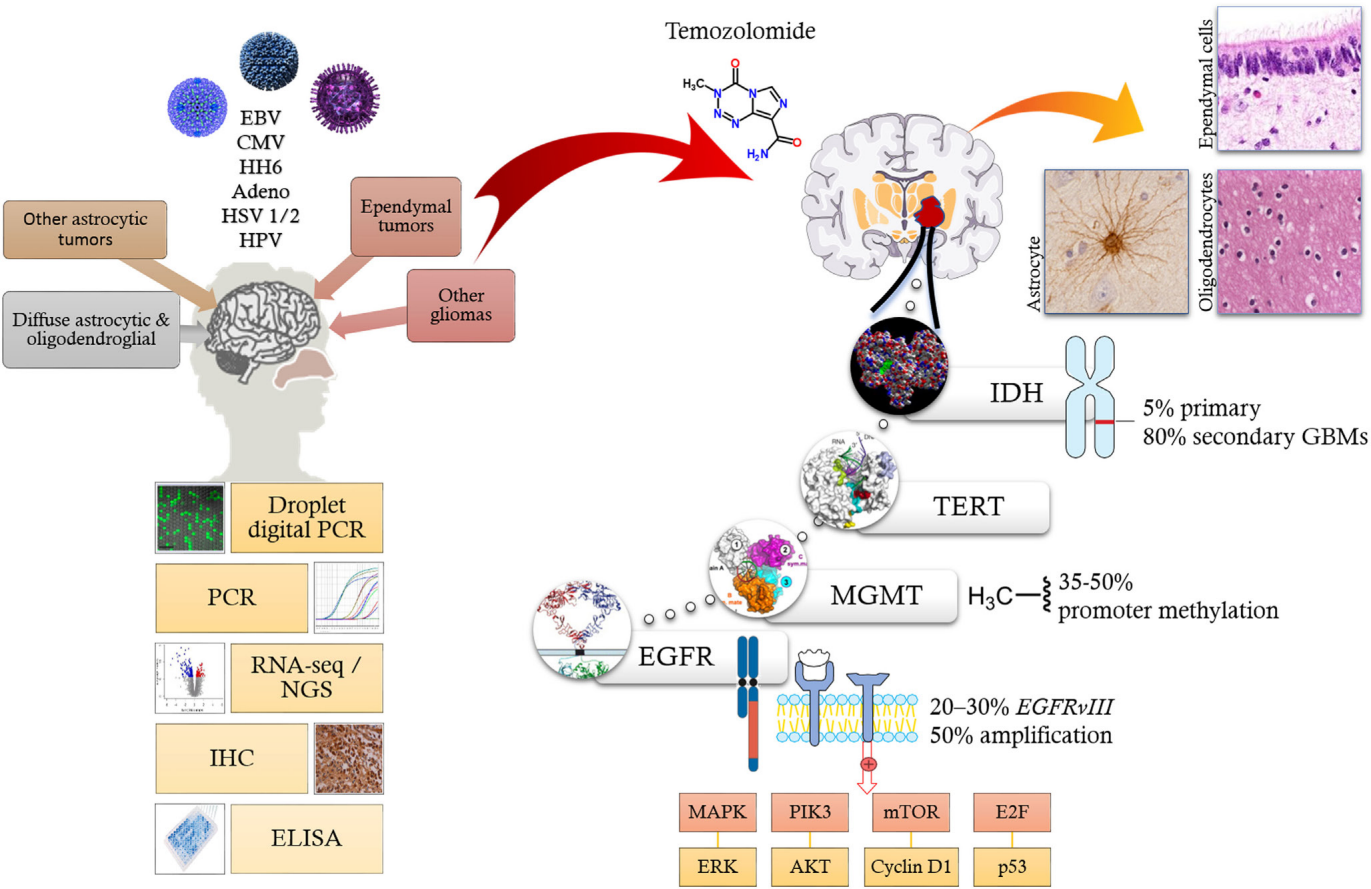
Oncoviruses, such as EBV, CMV, HH6, adenovirus, HSV 1/2, and HPV (top left) have been linked to CNS tumors like gliomas based on various molecular biology techniques (bottom left). Current literature implicates multiple molecular pathways facilitating the formation of both low-grade and high-grade-gliomas. Signaling aberrations mainly involve *EGFR* amplification; metabolic alteration *via* IDH1; manipulation of cell cycle, DNA repair and apoptosis *via* tyrosine kinase signaling ERK/ATK, cyclins, E2F, and p53; epigenetic silencing of DNA repair genes like *MGMT*; and activation of telomerases *via* mutations of *TERT* gene. Alkylating agents such as temozolomide alkylate/methylate, the DNA on guanine residues inducing DNA damage thereby induce apoptosis. Abbreviations: EBV, Epstein–Barr virus; CMV, cytomegalovirus; HH6, human herpes virus 6; HSV 1/2, herpes simplex virus type 1 and 2; HPV, human papillomavirus; PCR, polymerase chain reaction; RNA-Seq, RNA sequencing; NGS, next-generation sequencing; IHC, immunohistochemistry; ELISA, enzyme-linked immunosorbent assay; IDH, isocitrate dehydrogenase; TERT, telomerase reverse transcriptase; MGMT, O-6-methylguanine-DNA methyltransferase; EGFR, epidermal growth factor receptor; MAPK, mitogen-activated protein kinase; PIK3, phosphatidylinositol 3-kinase; mTOR, mechanistic target of rapamycin; ERK, extracellular signal-regulated kinases.

Recent advances in molecular profiling of brain tumors has led to better disease stratification by allowing a more clear distinction between the low-grade and high-grade gliomas (GBM) (10). As a result, the 2016 WHO classification of glial tumors has integrated the classical tumor morphology with genomic alterations derived from molecular profiling studies (11, 12). Most gliomas harbor molecular alterations disrupting key signaling pathways involved in regulation of cell growth (e.g., receptor tyrosine kinases, MAPK/ERK PIK3CA/AKT/PTEN signaling pathways), cell cycle/DNA repair/apoptosis (e.g., retinoblastoma/E2F/p53), metabolism [e.g., isocitrate dehydrogenase (IDH1)], chromatin, and telomere length (13). Among the most relevant genetic alterations affecting GBM are mutations of the *IDH* gene that may be linked to survival (14, 15). The enzyme catalyzes the oxidative decarboxylation of isocitrate to α-ketoglutarate and reduces NAD(P)+ to NAD(P)H (16). IDH has two isoforms (IDH1 and IDH2) of which mutations in *IDH1* are the most common (Figure 1). *IDH* gene mutations are present in only 5% primary and approximately 80% of secondary GBMs (14).

Epidermal growth factor receptor (EGFR) is commonly overexpressed in GBM, most frequently due to *EGFR* gene amplification and/or the *EGFR variant III* deletion mutation (EGFRvIII). *EGFR* gene amplification is observed in approximately 50% of GBMs, whereas *EGFRvIII* (Figure 1), a constitutively active truncated form of the EGFR protein that lacks the extracellular domain, occurs in 20–30% of cases (11, 17–19). Indeed, targeted inhibition of EGFR or the tumor-specific EGFRvIII holds therapeutic promise and several clinical trials with specific tyrosine kinases as well as monoclonal antibodies are ongoing (20, 21).

O^6^-methylguanine-DNA methyltransferase (MGMT) is an enzyme that is involved in DNA repair. MGMT promoter methylation is commonly detected in GBMs (~35–50%), particularly among the secondary GBMs (10, 12). Epigenetic silencing of the MGMT DNA-repair gene by promoter methylation compromises DNA repair and has been associated with longer survival in patients with glioblastoma who receive alkylating agents including temozolomide (Figure 1) (22–24).

Telomerase reverse transcriptase (TERT) is an enzyme that is responsible for adding nucleotides to telomeres. Telomerases are usually inactive in the adult normal cells, but can be reactivated (e.g., by mutations) in various cancers to promote oncogenesis (11). TERT gene mutations in GBMs are activating (usually in the TERT promoter region) (11). TERT gene mutations are particularly common in primary GBMs (Figure 1) (11, 14). Several therapeutic strategies for the inhibition of telomerases have been attempted (25).

In addition, GBMs are frequently affected by the various copy number aberrations (CNA). These involve gains at chromosomes 7 (EGFR/MET/CDK6), 12 (CDK4 and MDM2), and 4 (PDGFRA), while deletions are commonly observed at chromosomes 9 (CDKN2A/B) and 10 (PTEN) (13). A subset of GBMs may also have genetic alterations of *PIK3CA, PIK3R1, NF1*, and *RB1* genes (13, 19).

Along with improved understanding of the role of cells in the tumor microenvironment (e.g., reactive astrocytes, activated macrophage, and glioma stem cells), micro RNAs, and long non-coding RNAs in glioma progression (26–28), the above genomic and molecular changes are thought to be of growing importance in the diagnosis, development, classification, and therapy of gliomas. However, what actually triggers these molecular changes and oncogenesis in brain tumors remains poorly understood.

### Possible Viral Etiology of Gliomas and Scope of Review

Although little is known about the etiology of GBM or other gliomas, increased risk has been observed following exposure to ionizing radiation (8) or chemical agents or through genetic predisposition (e.g., germline *TP53, NF1*, and *NF2* mutations) in a small proportion of the patients with GBM (e.g., Li–Fraumeni syndrome, neurofibromatosis type 1 and type 2) (8). More recently, increasing emphasis has been placed on a viral etiology of gliomas as they might serve as oncomodulators (29, 30). Oncomodula-tion refers to the ability of viral proteins and non-coding RNAs to promote oncogenic processes without direct oncotransformation, but through disturbances in various intracellular signaling pathways (30).

Viruses may contribute toward oncogenesis and tumor development in humans by inducing immunosuppression, modifying host cells through inducing oncoproteins, or altering the expression of host cell proteins at viral integration sites (29, 31). Pagano and colleagues have recently reviewed the most common cancer-causing viruses (31). Viruses such as human papillomavirus (HPV) and human cytomegalovirus (CMV)—also known as human herpes virus-5 are strongly linked to the etiology and progression of cervical and colorectal cancers, respectively (32, 33). Several viruses have been linked to the etiology of brain tumors including CMV and other herpes viruses, such human herpes virus 6 (HHV-6 or roseolovirus), John Cunningham Virus (JCV; a polyomavirus); adenoviruses and Simian virus 40 (SV40), and others (30, 34). However, in the case of brain tumors, there is contradictory and/or controversial evidence linking many of these viruses, especially CMV—a ubiquitous herpes virus (32, 35). Because of its affinity for glial cells and its ability to reduce apoptosis, increase cell invasion, activate telomerase, and enhance angiogenesis in tumor cells (36, 37), several studies have investigated the role of CMV in glioma etiology. The first-ever study by Cobbs et al. in 2002 reported that CMV gene products and nucleic acids were present in all 27 glioma samples investigated, without being detected in other brain tissue (38). Despite confirmatory reports from other research groups (39, 40), recent conflicting reports showing no association of CMV in brain tissues (35, 41) have cast doubt on the role of CMV in brain tumors.

While the majority of the literature concerning viruses in glioblastoma thus far had focused on CMV, more recently attention has shifted to another potential oncovirus, EBV, and its role in the etiology of gliomas (Figure 1). In this review article, we will focus on providing a comprehensive review of the literature pertaining to EBV in gliomas and discuss the possibilities of this virus being causative, simply associative, or even an experimental artifact has been suggested by some recent highly sensitive “*state of the art*” next-generation sequencing-based virome detection assays.

### EBV and Tumorigenesis

Epstein–Barr virus, named after Michael Anthony Epstein and Yvonne Barr is also known as HHV-4, and was the first recognized human oncovirus (42). It belongs to the group of gamma-herpes viruses and is present in more than 90% of the human adult population who largely remain asymptomatic (43) with the main mode of transmission being *via* salivary and genital fluids (44). EBV, along with other herpes virus family members, is responsible for infections widely spread in the general population. Exposure mostly occurs in childhood or young adulthood followed by lifelong persistence of the virus. Thus, EBV has two distinct life cycles in humans: an acute lytic cycle, during which the production of new virions occurs; and a latent form, in which the EBV remains “hidden” in the host. Although, EBV typically remains in memory B-cells, in a latent phase, it may also be detected in epithelial cells (oropharynx) as well as in certain subsets of T and NK cells (44).

Epstein–Barr virus is a DNA virus whose genome is approximately 172 kb in length (44). Binding of its surface protein gp350 with CD21 receptor [also known as complement receptor 2 (CR2)] followed by viral glycoprotein gp42 interaction with cellular MHC class II molecules represents the major cellular fusion and entry mechanism into B-cells, whereas entry into epithelial cells is facilitated by viral protein BMRF-2 binding to cellular β1 integrins (44, 45). Subsequent to primary infection and replication within the lytic cycle, most of EBV genes are turned off as the virus switches to the latent phase (29).

During latency, EBV genome circular DNA resides in the cell nucleus as an episome and is copied by cellular DNA polymerase. In latency, only a portion of EBV’s genes including the six EBV nuclear oncoproteins (EBNA1, -2, -3A, -3B, -3C, and -LP) and the three latent membrane proteins (LMP1, -2A, and -2B), as well as several non-coding RNAs (EBERs and miRNAs) (46–49) are expressed in one of three patterns, known as latency programs (namely latency I, latency II, and latency III). Each latency program, therefore, leads to the production of a limited, distinct set of viral proteins, and viral RNAs. As mentioned EBV can latently persist within B cells and epithelial cells, but different latency programs are possible in the two types of cell (50, 51). In cases of EBV-associated cancers, there is differential expression of viral latency genes. However, emerging evidence suggests that of these, LMP1 is a major EBV-oncoprotein, as it provokes a multitude of effects enhancing cell growth, protecting cells from apoptosis, promoting cell motility and angiogenesis, and it is frequently expressed in EBV-linked human oral carcinomas (52–54).

Severe infections with EBV can cause infectious mononucleosis (glandular fever), and its latent state can revert (i.e., reactivate virus) to yield multiple lymphoid and epithelial malignancies, including B-cell lymphomas (Burkitt’s lymphoma, Hodgkin’s lymphoma (HL), post-transplant lymphoproliferative disorder), various T-cell/NK lymphoproliferative disorders, undifferentiated nasopharyngeal, and gastric carcinomas (55–57). Recent investigations including three from the Middle East, suggest that EBV is also present in around 40% of human breast malignancy where its occurrence is linked with more aggressive pheno-types (58–64).

Epstein–Barr virus can induce several molecular signaling changes in tumors such as those described in HL and undifferentiated nasopharyngeal carcinoma. Approximately 50% of HLs are associated with EBV infection, particularly its lymphocyte-depleted and mixed-cellularity variants. Reed–Sternberg (RS) giant cells represent characteristic B lymphocyte transformed neoplastic cells in HL, which are infected by EBV. Activation and survival of these cells are largely dependent on NF-ĸB upregulation through the intimate interaction of CD40 receptor and LMP1 oncoprotein of EBV (65). In addition, several signaling pathways may also be upregulated by this interaction, including MAPK/ERK, PIK3CA/AKT, JAK/STAT, and Notch pathways (44). EBNA-1 is another important EBV product that is required for the replication and maintenance of EBV genome in cancer cells (44). Thus, in case of undifferentiated nasopharyngeal carcinoma, LMP1, LMP2, and EBNA1 products of EBV are actively involved in promotion of cell growth and anti-apoptotic effects in neoplastic cells (66), while LMPA2A is responsible for preventing the differentiation of the epithelial cells (46). All these EBV products are also involved in other processes (e.g., immune evasion, metastasis) that contribute a highly aggressive phenotype and poor clinical outcome of undifferentiated nasopharyngeal carcinomas (66). Of note, EBV presence has been well documented in several other cancers, including breast, prostate, oral, and salivary gland carcinomas (67–70).

## EBV and Gliomas

Although the role of EBV in B-cell lymphomas and nasopharyngeal carcinomas is well-defined, its role in gliomas is only recently being explored. EBV, whose main latent reservoir is thought to be B-cells in the bone marrow, is also known to be present in the brain. Although rare, EBV infections can be found in the CNS especially in immunocompromised patients as exemplified by a case of EBV-induced encephalitis (71). Further, EBV is causally associated with a number of other CNS disorders [infectious mononucleosis, acute encephalitis, acute cerebellar ataxia, demyelinating disease, myelitis or meningitis, and some CNS neuropathies (72)]. The major cellular receptor for EBV, compliment receptor 2 (CR2) appears to be present on astrocytes (73) facilitates entry to infect astrocyte cell lines (74), and leads to increased proliferation. Importantly, primary CNS lymphomas (e.g., diffuse large B-cell lymphomas and lymphoid granulomatosis) are frequently EBV-positive (75). Thus, the fact that EBV is also thought to be the causative agent for primary CNS lymphomas in immune-deficient patients has led to its investigations in other brain tumors including gliomas.

### Literature Survey of EBV in Gliomas

In this section, we provide a detailed review of the key studies on EBV in gliomas (see Table 1). We searched the PubMed/MEDLINE databases using the following key words, such as glioma(s), glioblastoma multiforme, brain tumors/cancers, EBV, and neurotropic viruses. Our literature search was not time limited.

**Table 1:** Selected examples of studies investigating Epstein–Barr virus (EBV) in gliomas.

Reference	Glioma type	Sample size/tissue sampled	Methodology	Main findings
Strojnik et al. (34)	High-grade	45 adult patients, tumor biopsy	*ebna* RT-polymerase chain reaction (PCR)	3/45 (6.7%) positive
Wrensch et al. (76)	High-grade	57 adult patients, serum analysis	Enzyme-linked immunosorbent assay (ELISA) for IgG in sera	86% positive
Poltermann et al. (77)	High-grade	35 patients, serum analysis	ELISA for IgG in sera	90% positive
Zavala-Vega et al. (78)	High-grade	21 patients, tissue biopsy	Detected latent membrane proteins (LMP-1) by immunohistochemistry and EBER expression by *in situ* hybridization, RT-PCR	6/21 (28.6%) positive
Fonseca et al. (79)	Low-grade and high-grade	75 patients, tissue biopsy	EBV using PCR with confirmation using direct sequencing	6/11 (55%) low-grade positive3/22 (13.6%) high-grade positive
Cheng-Te Major Lin et al. (41)	High-grade	19 patients, formalin-fixed glioma tissue	EBV *lmp1* DNA with multiplex droplet digital PCR	4/19 (21%) positive
Neves et al. (80)	Pilocytic astrocytoma	35 children, tissue biopsy	RT-PCR, LMP1 by immunohistochemistry	9/35 (26%) positive by PCR, but none by immunohistochemistry
Cimino et al. (81)	High-grade	21 patients, tissue biopsy	Next-generation sequencing/PCR/*in situ* hybridization	5/21 (24%) positive, but none by *in situ* hybridization
Strong et al. (35)	High-grade	170 patients, tissue biopsy	Next-generation sequencing/RT-PCR	None positive
Cosset et al. (82)	High-grade	20 patients, tissue biopsy/serum analysis	PCR	None positive
Khoury et al. (83)	Low- and high-grade	215 patients/tissue biopsy	RNA-Seq database analyses	None positive
Hashida et al. (84)	High-grade	39 patients/tissue biopsy	PCR analyses of *LMP1* gene	None positive

Several, but not all, of the studies conducted across different geographical locations, such as North America, South America, Europe, and Japan, have shown a positive association of EBV in patients with gliomas (Table 1). Recently, Stojnik et al. (34) studied the presence of EBV, along with HSV-2, HHV-6, and one human enterovirus (hEV) in high-grade gliomas in 45 adult patients (12 with grade III and 33 with grade IV) at the University Clinical Centre in Maribor, Slovenia. Glioma tissue samples were obtained either from tumor biopsies (19/45) or following surgical tumor reduction (26/45) from patients with a median age of 60 years (ranging from 22 to 86 years). Tissue was either used within 24 h for assaying of viral genes by rt-PCR (in the case of EBV, a 166 bp fragment of the *ebna* gene was amplified). Serum analyses of C-reactive protein (CRP) was measured for all patients (24 whom were females) and 30/45 patient samples were also analyzed for specific antibodies for each of the viruses by enzyme immunoassays and complement fixation. PCR studies of gliomas revealed only 3/45 patients were positive for EBV *ebna* gene: a 66-year-old male with GBM located in the left temporal and parietal lobes; a 68-year-old female with GBM located in the right temporal and parietal lobes; and a 77-year-old male with GBM located in the right temporal, parietal, and occipital lobes. Common features were that all samples were attained following craniotomy and surgical tumor reduction. Importantly, all three EBV+ patients had grade IV gliomas (GBM) and no virus was detected in any of the 12 grade III gliomas, implying this virus is preferentially associated with most aggressive CNS tumors. However, none of the patients were found to be seropositive for EBV antibodies (34). This was in contrast to an earlier report by Wrensch et al. (76), who used serological IgG antibody binding using ELISA assays to demonstrate that about 90% of their GBM patients, from the USA, San Francisco Bay Area Adult Glioma Study from 1991 to 1995, were seropositive for EBV (76). Another study conducted by Poltermann et al. (77) showed the presence of IgG antibodies to EBV in serum of 89% (64/72) of patients with glial tumors (*n* = 35), meningiomas (*n* = 31), and acoustic schwanommas (*n* = 6) though this was not considered significantly different to antibody levels in the general population (77).

Strojnik et al. (34) also found HHV-6 in 2/45, HSV2 in 1/45, and hEV in 1/45 glioma tissue samples tested. All positive tests were in grade IV gliomas but of varying origin. However, viral copy numbers for all viruses, including EBV, detected in glioma tissue samples were generally very low (mostly below 2 copies per 5 µL DNA with only the 66-year-old male with EBV having a copy number of 27 copies per 5 µL DNA). Again, none of the patients’ positive for HHV-6 or HSV2 in glioma tissues developed antibodies in serum samples though five positive results for HSV2 antibodies were noted even in the absence of virus in the tumor samples. Furthermore, the presence of adenoviruses, HSV-1, CMV, and VZV was not confirmed in any of the 45 tissue samples studied (34).

Another recent study by Zavala-Vega et al. (78) reported on presence of EBV, along with CMV and HSV1/2 in Mexican patients with GBM. They performed a retrospective study using brain tissue from 21 adults aged on average 52 years (range 23–83 years). To indicate EBV infection, they detected LMP-1 by immunohistochemistry and EBER expression by *in situ* hybridization in 6/21 (28.6%) of patients. Mixed infections of EBV and HSV-1/2 were noted in 4/21 patients (19%), whereas EBV and CMV in 5/21 (23.8%) patient samples. A particular limitation of this study was that IgG and IgM antibody levels could not be determined in patients with viral infections as this was a retrospective study based on paraffin-embedded tissue samples only. However, the value of measuring antibody titers may not correlate with disease as antibodies produced in the case of the related CMV during early stages of infections have a protective effect, thereby preventing viral reactivation and subsequent development of glioblastoma (85).

A study by Fonesca et al. (79) aimed to screen 75 primary glioma biopsy specimens from a cancer centre in Rio de Janeiro, Brazil, for the presence of EBV using PCR with confirmation using direct sequencing. To detect EBV in tumor samples, a 288 bp fragment of EBV bam region was amplified and later sequenced to confirm viral DNA in GeneBank data sets. Using this strategy in fresh frozen tissue samples, 11/75 gliomas (14.7%) were positive for EBV with the majority being low-grade gliomas (6/11), followed by 2/11 for grade III, oligoastrocytoma (1/11), ependymoma (1/11), and only 1/11 being grade IV (GBM). These results are in contrast to the study from Slovenia where only high-grade gliomas were positive for EBV (34). In addition, Fonesca et al. (79) also found EBV in one oligoastrocytoma and one ependymoma, but none at all in other CNS tumors including two non-HL—a tumor type in which EBV association has been reported previously (75). The amplified EBV gene sequences obtained from gliomas were well matched with published EBV genome sequences with an identicalness rate of 95.5% implying that EBV virus was indeed present in these samples.

Cheng-Te Major Lin et al. (41) used multiplex droplet digital PCR (ddPCR)—a highly precise diagnostic tool that enables the absolute quantification of target DNA in a high throughput setting—to show positivity of EBV *lmp1* DNA in 4/19 (21.1%) of formalin-fixed paraffin-embedded (FFPE) GBM samples and not in any controls. Samples were obtained from the George Washington University Hospital and the National Institutes of Health, USA. Interestingly, two GBM tumor specimens were positive for both HHV-6B and EBV indicating that the possibility of multiple viral infections being associated with GBMs.

Pilocytic astrocytoma of the cerebellum is one of the most common pediatric brain tumors. In FFPE tumor samples analyzed by two different PCR methodologies and immunohistochemistry, EBV was detected by PCR in about 30% of these tumors (9/35) from patients with an average age of 15.5 years; however, none of the samples were positive for EBV by immunohistochemistry (anti-LMP1 antibody) (80). Most of the astrocytoma (33/35) was of low-grade malignancy. This study suggested that EBV was the most frequent herpes virus found in pilocytic astrocytoma though at levels apparently too low to be considered responsible for tumor induction (80).

Because polymerase chain reaction (PCR) analyses and viral-specific immunohistochemical assays are biased in that only selected or targeted genes or proteins of viruses are investigated in tumors, more state-of-the art methods with high sensitivity that may avoid these bias are being used to detect infectious agents in tumors. A less biased approach would be to fully sequence brain tumors and search for any EBV virome nucleic acid sequences present. One such methodology that allows this rather unbiased approach is next-generation sequencing (NGS)—a non-Sanger-based high-throughput DNA sequencing technology. There are a number of different NGS platforms, a detailed discussion of which is beyond the scope of this article, but the reader is referred to some recent review (86–88). Nonetheless, in all NGS platforms sequencing of millions of small fragments of DNA in parallel is followed by bioinformatics analyses to piece together these fragments and mapping the individual reads to the reference genome. NGS can be used to sequence entire genomes or constrained to specific genes or regions of interest. Recently, NGS studies have been used to study the presence of EBV sequences in gliomas (71, 81).

A NGS study by Cimino et al. (81) examined viral sequences in 21 high-grade gliomas (mostly glioblastomas) at the University of Washington, St Louis, MO, USA. Unmapped sequencing reads, obtained from FFPE samples, identified EBV in 5/21 (24%) of high-grade gliomas (all GBM). They also found one case of Roseolovirus, but no CMV in any of their glioma tissues. However, further examination of the four of EBV-sequence-positive tumors for virus by *in situ*-hybridization failed to detect EBV-encoded RNA implying that EBV in malignant high-grade gliomas might be transcriptionally inactive and more characteristic of a dormant state that could also be present in the general population (81). However, since the authors examined only one non-coding EBER RNA, the possibility that other EBV RNAs may be produced still remains unexplored.

Contrary to the findings of Cimino et al. (81), a more recent and very comprehensive NGS study by Strong et al. (35) suggested that no major viruses were associated with high-grade gliomas. These authors undertook a large-scale virome assessment in publically available The Cancer Genome Atlas (TCGA) sequencing data sets for 157 primary glioblastomas (GBM) and 13 recurrent GBM as well as whole genome sequencing (WGS) data sets for 51 primary GBM, and 10 recurrent GBM. Finally, they also analyzed fresh frozen tissue from three primary GBM samples (one from a patient at the Louisiana Cancer Research Consortium and two samples from the commercial supplier BioServe, USA). In this comprehensive and detailed study, the authors aimed to address many of the major experimental concerns in detecting viruses in tumor tissues (35). For instance, to account for heterogeneity within GBM tumor mass that might give rise to differential transcriptome profiles (89), they used data sets from 92 MRI-localized biopsies from either the core or margins of multiple GBM patients; and to account for the possibility that viruses may lay hidden within cancer stem cells, they also analyzed RNA-seq data sets from a cohort of short-term glioma stem cell cultures freshly isolated from nine patients with primary GBM. Despite these precautionary measures, as well as running NGS experiments at low viral read thresholds (that could have been associated with increased risk of low-level contamination), no major virus associations could be identified. However, in their attempt to account for the possibility that viruses infecting brain tissue become transcriptionally dormant and thus avoid detection in RNA-seq data sets, they also looked at WGS data sets for virome assessment. Analyses of the virus at the DNA level did show low level presence of EBV DNA in samples (at viral reads below 40) from 9 primary GBM and 6 matched blood samples as well as 3 recurrent GBM each from the TCGA and WGS data sets with only one having a moderate EBV viral read of 1,454. However, the presence of EBV DNA in the case of the three recurrent GBM from WGS data could not be validated by the corresponding RNA-seq data. As true, EBV association would normally lead to much higher viral reads (>10 for RNA-seq and >1,000s for DNA-seq) and given the presence of EBV in blood and tumor specimens was roughly equivalent, the authors concluded that the detected EBV likely originated from infiltrating EBV-infected B-cells and/or from possible library or sequencing sample cross-contamination.

Similarly, they also dismissed low-level viral reads of several other viruses in gliomas, as likely artifacts or non-pathological incidental infections. For example, they noted that all of the sporadic low-level CMV reads were found to map to the immediate early promoter intimating that they likely originated from laboratory expression vector contamination. In addition, human herpes virus 6 and 7 aligned viral reads were likely false-positives due to their homology with human telomeric-like repeats (35). These data argue against associations between most known viruses and GBM or meningiomas, but interestingly, the authors highlighted that the most robust virus findings were the detection of HPV and hepatitis B in the occasional low-grade gliomas. Thus, although these findings cast doubt on EBV association in gliomas they rather, open the door for the further in-depth studies on the possible association of HPV and hepatitis B, two viruses that have received little attention in CNS tumors including gliomas.

Several other studies have reported on the complete absence of EBV in gliomas. Cosset et al. (82) studied 20 GBM biopsies including the corresponding patient serum, where available, by standard clinical diagnostic methods (semi-qPCR) for the presence of the following common neurotropic viruses: CMV, EBV, HSV, HHV6, MeV, PeV, JC virus, EV, and VZV. Although some biopsies were associated with a type I IFN-response, none of the above-mentioned viruses were detected in any sample of GBM or of three other low-grade gliomas, one oligodendroglioma, two meningiomas, one ependymoma, and one oligoastrocytoma (82). Similarly, Khoury et al. (83) reported no EBV or any other virus after screening of TCGA malignant tumors including low- and high-grade gliomas on which RNA-Seq data were available. They showed no evidence of transcribed viral elements in any of the low-grade gliomas and glioblastoma multiforme. Further, a study by Hashida et al. (84) in Japanese subjects with GBMs failed to detect EBV in tumors using real-time PCR analyses of *LMP1* gene. However, these authors did show the presence of high risk HPV16 and HPV18 in 21% (8/39) of the GBMs studied—results that are consistent with the findings of Vidone et al. (90) in Italian glioma patients and reaffirmed in the NGS study of Strong et al. (35) discussed above (see also Table 1).

## Perspectives and Concluding Remarks

It is clear from the studies examining EBV in gliomas conducted thus far that, as is the case with other viruses like CMV, there are discordant results on viral association in these malignancies. Reasons for these discordant findings may lie within population/geographic differences, individual genetic variability, inherent heterogeneity of gliomas, variations in samples including anatomical location from which tumor specimen was removed, differences in the actual viral genes probed, as well as sensitivity and precision of the methodologies used. In addition, differences in processing or preparation of samples (such as section thickness, fixation conditions, and antibody dilution) and difficulties with paraffin-embedded tumor samples may have caused the observed discrepancies (32, 91). Are these variables really the explanation? Probably, in part but surely, an ideally robust association of EBV in gliomas would have resulted in sufficiently high viral levels to the extent that the effects of many of the above variables would be minimal or at least mitigated to some extent. However, a few studies have shown no virus and most have shown only low levels of the EBV either in the glioma tissue or as antibodies in serum including the recent elegant and comprehensive study by Strong et al. (35) that aimed to account for many of the concerns mentioned above.

Serological studies measuring EBV antibodies in glioma patients were also discordant. Given the fact that 90% of the population is carriers of EBV in its latent state, why are we not, therefore, detecting a similar proportion of seropositive tumor patients as the general population in all studies? For example, in one study, the risk of glioma patients being seropositive for EBV was less than the control population implying that the tumor may actually modulate EBV infections. The presence of lower levels of EBV in tumor than in control samples could also be explained if the virus was lost during tumor progression. Such a “hit and run” model has been proposed in HPVs (28, 92). There is also evidence indicating the presence of EBV antibodies early on may actually be protective in tumors (93). In any case, the high seroprevalence of EBV in controls makes it difficult to make a firm association based on the serum antibody data presented for EBV in gliomas.

Thus, can we really exclude a clinical role of EBV in gliomas based on these findings or could the relatively low levels of EBV, as generally reported in gliomas, still lead to gliomagenesis and/or oncomodulation? A recent report by Shumilov et al. (94) suggests that EBV might exert some of its oncogenic effects, such as inducing centrosome amplification and chromosomal instability, without having to establish a chronic infection, thereby conferring a risk for development of tumors that do not necessarily carry the viral genome (94).

Lytic replication, the process by which viral progeny is produced, is a strong risk factor for EBV-associated tumors (31). This process activates cellular cancer-associated changes such as chromosomal instability, but lytic replication also leads to cell death rendering the link between replicating cells and oncogenesis not so obvious. Shumilov et al. (94) presented the data that removed this conceptual difficulty by showing that the EBV virions themselves conferred the risk induced by lytic replication to non-replicating cells, i.e., the effects of EBV virions extended to EBV-negative cells. Thus, their paradigm-changing study implies that EBV could be a risk factor for the development of gliomas without being present in the tumor. If others confirm these findings, then this would fundamentally change our view of the role played by EBV in tumors and offer a more rational explanation for the near absence of EBV in gliomas reported in several of the studies reviewed herein.

Since direct viral association studies have generally been discordant, another approach to establish viral association with tumors has been to study the role of antiviral therapies on disease (91). A recent report has suggested that glioma patients at the Karolinska University Hospital receiving 6 months of antiviral therapy as an add-on to standard radiation and temozolamide therapy exhibited marked increases in survival rates (95), though the study design and mathematics used have been questioned (23, 95). Some other studies, but not all, have also shown improved outcomes in cancer patients on antiviral therapy (32, 96). However, while the rates of many AIDS-associated malignancies have been declining with the use of highly active anti-retroviral therapy, the rates of EBV-positive Burkitt’s and HL in this population have not declined (97, 98). These data may imply that the oncogenic effects of EBV—at least in B-cell lymphomas—are not affected by antiretroviral drugs. It should, however, be noted that while viral therapy may improve clinical outcome in some cases, it does not necessarily imply a viral cause as survival benefit might be explained by secondary or “off-target” effects of the therapy alone unrelated to viral infection.

In contrast, there is also evidence to suggest that prior exposure to stress and/or immunodeficient status induced by therapies may actually predispose patients to EBV-induced oncogenesis. For example, Zakaria et al. (99) described a patient who within 2 months of undergoing radio-chemotherapy for glioblastoma developed an EBV-positive primary diffuse large B-cell CNS lymphoma (99). These findings suggest that probably the immunosuppression and/or stress induced by the treatments for GBM, or even co-morbidities, can lead to EBV reactivation.

A corollary of this is the idea that stress resulting from co-infections may also be important in viral reactivation and oncogenesis. Although the low levels of EBV infections reported in gliomas, by themselves may not be sufficient, they likely require additional stress-causing risk factors, such as the co-presence of other oncoviruses, to influence oncogenesis or oncomodulation. Thus, latent EBV viruses may be reactivated when cells experience co-infection with, for example, CMV or HSV1/2, as has been reported in some glioma studies (78).

In this regard, a preventative vaccine against EBV and/or co-infecting agent may be useful. Vaccines against specific viruses may, therefore, offer a more targeted approach for association studies and clinical therapy [for review see Cohen (100)]. For example, an EBV vaccine has been tested (in a phase I clinical trial) on Chinese nasopharyngeal carcinoma patients to determine the safe and immunogenic dose (101). In that study, it was concluded that the vaccine is both safe and immunogenic, thus paving the way for further clinical testing of the EBV vaccine that may be of clinical benefit in EBV-positive tumors including glioma patients. The first prophylactic EBV vaccine based on virus-like particles (VLPs) that mimic the structure of the EBV virus, but lack its genome has also been reported to be effective in preclinical models (102) and may represent a safer alternative.

Finally, could it be that by looking for EBV and other herpes viruses like CMV, in gliomas we might have been focusing on the wrong viruses? Recent NGS sequencing data seems to suggest that most of the viruses especially CMV are completely absent from gliomas and many of the positive associations reported are likely artifactual as they may be rationally explained otherwise [e.g., high homology of detected viral sequences to host as in the case of chromosomal telomere repeats (35)]. The reported low level presence of EBV does not completely rule it out from being associated with oncogenesis or oncomodulation in gliomas [indeed, it may not even need to be present to exert its effects as suggested by the study of Shumilov et al. (94)], but recent reports suggest that HPV infection might be more robustly associated with some gliomas. Thus, additionally more detailed and comprehensive studies are needed to fully implicate EBV and/or other viruses such as HPV in having a direct association in gliomagenesis and oncomodulation. Understanding the role of EBV and other oncoviruses in the etiology of gliomas, that generally have a poor prognosis, would likely open up new avenues for the treatment and management of these, often fatal, CNS tumors.

## Author Contributions

SA and A-EM conceived the review. SA and SV searched the literature. SA, SV, FC, and A-EM critically appraised the literature, wrote and approved final version of the manuscript.

## Conflict of Interest Statement

The authors declare that the research was conducted in the absence of any commercial or financial relationships that could be construed as a potential conflict of interest.
